# Functional characterization of *HNF4A* gene variants identify promoter and cell line specific transactivation effects

**DOI:** 10.1093/hmg/ddae027

**Published:** 2024-03-03

**Authors:** Alba Kaci, Marie Holm Solheim, Trine Silgjerd, Jorunn Hjaltadottir, Lorentze Hope Hornnes, Janne Molnes, Andre Madsen, Gry Sjøholt, Christine Bellanné-Chantelot, Richard Caswell, Jørn V Sagen, Pål R Njølstad, Ingvild Aukrust, Lise Bjørkhaug

**Affiliations:** Mohn Center for Diabetes Precision Medicine, Department of Clinical Science, University of Bergen, Haukelandsbakken 1, Bergen 5020, Norway; Center for Laboratory Medicine, Østfold Hospital Trust, Kalnesveien 300, Grålum 1714, Norway; Mohn Center for Diabetes Precision Medicine, Department of Clinical Science, University of Bergen, Haukelandsbakken 1, Bergen 5020, Norway; Department of Safety, Chemistry, and Biomedical Laboratory Sciences, Western Norway University of Applied Sciences, Inndalsveien 28, Bergen 5020, Norway; Mohn Center for Diabetes Precision Medicine, Department of Clinical Science, University of Bergen, Haukelandsbakken 1, Bergen 5020, Norway; Department of Safety, Chemistry, and Biomedical Laboratory Sciences, Western Norway University of Applied Sciences, Inndalsveien 28, Bergen 5020, Norway; Department of Medical Biochemistry and Pharmacology, Haukeland University Hospital, Jonas Lies veg 87, Bergen 5021, Norway; Mohn Center for Diabetes Precision Medicine, Department of Clinical Science, University of Bergen, Haukelandsbakken 1, Bergen 5020, Norway; Department of Medical Genetics, Haukeland University Hospital, Jonas Lies veg 87, Bergen 5021, Norway; Department of Clinical Science, University of Bergen, Jonas Lies veg 87, Bergen 5020, Norway; Department of Safety, Chemistry, and Biomedical Laboratory Sciences, Western Norway University of Applied Sciences, Inndalsveien 28, Bergen 5020, Norway; Départment of Medical Genetics, Sorbonne University, AP-HP, Hôpital Pitié-Salpêtriére, 21 rue de l'école de médecine, 75006 Paris, France; Exeter Genomics Laboratory, Royal Devon University Healthcare NHS Foundation Trust, Barrack Rd, Exeter EX2 5DW, United Kingdom; Mohn Center for Diabetes Precision Medicine, Department of Clinical Science, University of Bergen, Haukelandsbakken 1, Bergen 5020, Norway; Department of Medical Biochemistry and Pharmacology, Haukeland University Hospital, Jonas Lies veg 87, Bergen 5021, Norway; Mohn Center for Diabetes Precision Medicine, Department of Clinical Science, University of Bergen, Haukelandsbakken 1, Bergen 5020, Norway; Children and Youth Clinic, Haukeland University Hospital, Haukelandsbakken 1, Bergen 5021, Norway; Mohn Center for Diabetes Precision Medicine, Department of Clinical Science, University of Bergen, Haukelandsbakken 1, Bergen 5020, Norway; Department of Medical Genetics, Haukeland University Hospital, Jonas Lies veg 87, Bergen 5021, Norway; Department of Safety, Chemistry, and Biomedical Laboratory Sciences, Western Norway University of Applied Sciences, Inndalsveien 28, Bergen 5020, Norway

**Keywords:** HNF4A-MODY, functional studies, transactivation, DNA binding, nuclear localization

## Abstract

Hepatocyte nuclear factor-4 alpha (HNF-4A) regulates genes with roles in glucose metabolism and β-cell development. Although pathogenic *HNF4A* variants are commonly associated with maturity-onset diabetes of the young (MODY1; HNF4A-MODY), rare phenotypes also include hyperinsulinemic hypoglycemia, renal Fanconi syndrome and liver disease. While the association of rare functionally damaging *HNF1A* variants with HNF1A-MODY and type 2 diabetes is well established owing to robust functional assays, the impact of *HNF4A* variants on HNF-4A transactivation in tissues including the liver and kidney is less known, due to lack of similar assays. Our aim was to investigate the functional effects of seven *HNF4A* variants, located in the HNF-4A DNA binding domain and associated with different clinical phenotypes, by various functional assays and cell lines (transactivation, DNA binding, protein expression, nuclear localization) and *in silico* protein structure analyses. Variants R85W, S87N and R89W demonstrated reduced DNA binding to the consensus HNF-4A binding elements in the *HNF1A* promoter (35, 13 and 9%, respectively) and the *G6PC* promoter (R85W ~10%). While reduced transactivation on the *G6PC* promoter in HepG2 cells was shown for S87N (33%), R89W (65%) and R136W (35%), increased transactivation by R85W and R85Q was confirmed using several combinations of target promoters and cell lines. R89W showed reduced nuclear levels. *In silico* analyses supported variant induced structural impact. Our study indicates that cell line specific functional investigations are important to better understand HNF4A-MODY genotype–phenotype correlations, as our data supports ACMG/AMP interpretations of loss-of-function variants and propose assay-specific *HNF4A* control variants for future functional investigations.

## Introduction

Hepatocyte nuclear factor-4 alpha (HNF-4A) is a transcription factor encoded by the *HNF4A* gene and plays a crucial role in the development, differentiation and function of the pancreas (β-cells), liver (hepatocytes), kidney (proximal tubules) and intestines (epithelium) [1–3]. It belongs to the nuclear receptor (NR) superfamily and comprises the following functional domains: transactivation domains AF-I and AF-II located in the N-terminus and C-terminus of the protein, a DNA-binding domain (DBD) including two zinc fingers and a hinge region, a dimerization/ligand-binding domain (LBD), and a C-terminal repressor domain [4]. The repressor domain, which inhibits the full transactivation potential of AF-II, is a unique feature among members of the NR superfamily. HNF-4A functions as a homodimer regulating the expression of target genes by a head-to-tail binding to specific and repetitive DNA response elements, referred to as DR1 [5, 6]. Heterodimerization by various HNF-4A specific isoforms, however, allows the regulation of a broader range of genes [7]. Further, the hydrophobic ligand binding pocket in the LBD domain facilitates the constitutive binding of fatty acids, allowing conformational change and interaction of co-activators [8] and subsequent HNF-4A activation.

**Figure 1:**
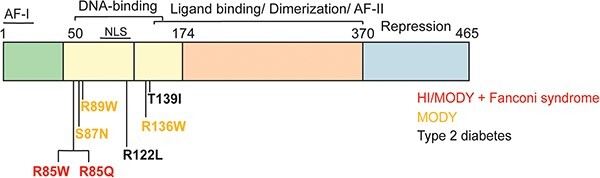
Schematic overview highlighting the HNF-4A protein sequence (isoform 2; NP_000448.3) and functional domains, as well as positions of HNF-4A variants investigated and associated with different glycemic phenotypes.

Rare variants in the *HNF4A* gene have been associated with a variety of clinical phenotypes. Briefly, loss-of-function variants cause Maturity-Onset Diabetes of the Young type 1 (MODY1; HNF4A-MODY, OMIM #125850); a monogenic form of diabetes characterized by progressive pancreatic β-cell dysfunction and impaired insulin secretion [9]. Some variants, however, result in a biphasic phenotype manifesting with neonatal hyperinsulinemic hypoglycemia (HH) and HNF4A-MODY later in life [10]. Further, variants affecting the R85 residue in HNF-4A have been reported in isolated families presenting with an atypical form of Fanconi renotubular syndrome (OMIM #616026), characterized by inadequate reabsorption of glucose, amino acids and low-molecular weight proteins in the proximal renal tubules of the kidney [11]. Moreover, common variants in *HNF4A* have been associated with a moderate increased risk for the development of type 2 diabetes (T2D) [12, 13]. As there have been few studies investigating naturally occurring *HNF4A* variants for functional impact, an explanation for the diversity in *HNF4A* genotype-phenotypes remains largely unknown. The availability of suitable and robust control variants in functional assays for evaluating variants’ effects for diagnostic purposes has therefore also been lacking. Due to this, there is a knowledge gap regarding *HNF4A* functional effect and its use in variant interpretation for precision medicine. This is in contrast to the more well characterized MODY3-associated *HNF1A* (HNF1A-MODY), for which guidelines have been developed to scale the functional impairment of variants [14]. To improve our understanding of the variant mechanistic effects underlying the *HNF4A* genotype-phenotype diversity, we hypothesized that this could be accomplished through investigations of the functional and structural consequences of seven variants located in the HNF-4A DBD and reported associated with either HNF4A-MODY, HNF4A-MODY and atypical Fanconi syndrome, or identified in rare T2D cases (Fig. 1 and Supplementary Table S1). Since HNF-4A isoform 2 (NM_000457.4), encoding a 474-amino acid protein, is the most highly expressed isoform in kidney and liver cells, variants in this isoform were investigated in terms of their transactivation activity using various promoter-linked reporters and cell line systems, for alterations in DNA-binding ability, and for protein expression and nuclear localization levels.

Aiming towards developing robust and meaningful functional HNF-4A assays, our study demonstrates the relevance of investigating promoter-specific transactivation in multiple relevant cell lines, supplemented with DNA binding and nuclear localization assays, to best evaluate *HNF4A* variant functional effects and to identify suitable assay control variants. Applying *in silico* structural tools for predicting possible variant induced structural effects was also a valuable supplement to our *in vitro* functional findings.

## Results

### Selection of variants

Five previously reported rare *HNF4A* missense variants classified as either variants of unknown significance (VUS) and including S87N and R122L, likely pathogenic (LP) and including R85Q, or pathogenic (P) and including R85W and R89W (Supplementary Table S1) [15], were functionally investigated. The remaining two variants were selected on the basis of expectations of a milder functional effect as being reported as either a low-penetrant HNF4A-MODY variant (R136W) [16, 17] or classified as benign with respect to MODY (T139I) [18]. Moreover, the selection of variants was also based on being located in close proximity (R85W, R85Q, S87N, R89W) in the HNF-4A DBD sequence, however being associated with different glycemic phenotypes in variant carriers.

### Altered transactivation of HNF-4A variants on *HNF1A* and *G6PC* promoters

**Figure 2:**
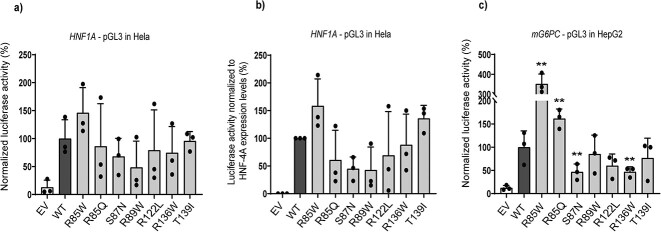
Transcriptional activity of HNF-4A variants investigated in transiently transfected (a) HeLa cells on pGL3-*HNF1A*, and in (c) HepG2 cells on pGL3-*G6PC* reporter plasmids. (b) Transcriptional activity after normalizing for protein expression levels in HeLa cells. For protein expression levels in HeLa/HepG2 see Supplementary Fig. S1. Empty vector (EV) was used as a negative control. Each bar represents the mean of nine readings ± SD; taken from three parallel readings from each of each of three independent experimental days. ^*^indicates *P* < 0.05.

The seven HNF-4A variants were first investigated for their ability to activate transcription in transiently transfected cell lines. HeLa cells were selected to avoid interference of endogenous HNF-4A. HNF-4A variant activity was investigated using an *HNF1A* promoter-linked luciferase reporter construct containing an HNF-4A recognition site (Fig. 2a). Although unable to reach statistical significance, R89W presented with lowest transactivation activity (< 50%), while S87N, R136W and R122L activities were ~70%–80% compared to WT activity. Near-normal activity was observed for T139I. Interestingly, R85W trended towards increased activity (~150%).

The relative protein expression levels of HNF-4A variants in cell lysates obtained from the transactivation assay (Fig. 2a) was further analyzed by SDS-PAGE and immunoblotting (Supplementary Fig. S1a–c). Normalization of activity obtained from the transactivation assay with variant protein expression levels did not dramatically alter the initial trend of the activity levels. Increased activity was still indicated for R85W (~160%), while still reduced, however non-significant, for R89W and S87N (43% and 45%, respectively) (Fig. 2b). Transactivation and protein expression data are also summarized in Table 1.

**Table 1:** Summary of functional investigation data on HNF-4A variants. The PS3_supporting or BS3_supporting ACMG scores were only applied when a variant was shown to be impaired (transactivation activity < 60% and DNA binding < 60%, compared to wild type) or showed no damaging effect (transactivation activity > 75% of wild type), respectively, in at least two functional assays by transactivation and/or DNA binding assay. The nomenclature used for the variants is based on the *HNF4A* transcript NM_000457.4 (HNF4a isoform 2). Values given in % of WT (100%); N/A, not available; N/R, not relevant.

HNF-4A variant	Transactivation *HNF1A prom* (HeLa)	Protein expression (HeLa)	DNA binding *HNF1A* (HeLa)	Nuclear localization (HeLa)	Transactivation *G6PC prom* (HepG2)	DNA binding *G6PC* (HepG2)	ACMG supporting functional evidence
EV	20	0	7	0	12	5	N/R
WT	100	100	100	100	100	100	N/R
R85W	146	92	35	240	352	10	-
R85Q	86	142	75	160	162	54	-
S87N	68	151	13	80	47	N/A	PS3_supporting
R89W	48	114	9	20	85	N/A	PS3_supporting
R122L	79	103	N/A	N/A	60	N/A	-
R136W	74	85	94	94	47	N/A	-
T139I	96	70	N/A	N/A	77	N/A	BS3_supporting

To confirm variant activity on a different HNF-4A target promoter and relevant cell line, we assessed the *G6PC* promoter-linked luciferase reporter in transiently transfected HepG2 cells (Fig. 2c). The low background activity by the empty vector (EV) indicated minimal interference by endogenous proteins on this reporter. The R85W variant demonstrated increased activity also on the *G6PC* promoter (> 350%). Variants S87N and R136W, however, demonstrated strongly reduced activity (47%) on the *G6PC* promoter. Activities of R122L, R89W and T139I were either 60% (R122L) or > 75% (Fig. 2d). Of note, normalization of transactivation by HNF-4A protein expression levels in transfected HepG2 cells was not suitable due to strong presence of endogenous HNF-4A protein detected by immunoblot analysis when using an HNF-4A specific antibody (Supplementary Fig. S1d–f).

### R89W and R85W differentially affects HNF-4A nuclear localization level

**Figure 3:**
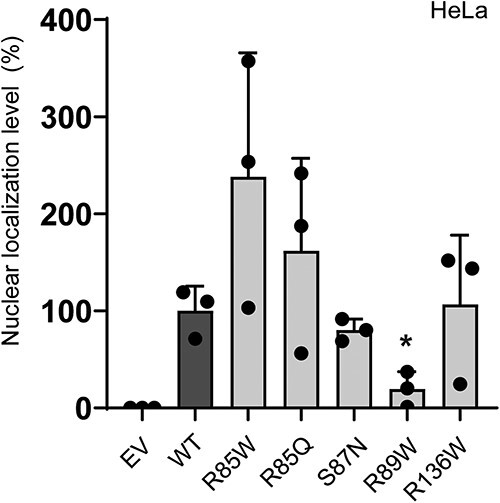
Effect of selected *HNF4A* variants on the nuclear localization of HNF-4A. Nuclear and cytosolic fractions were analyzed by SDS-PAGE and immunoblotting. Each bar represents the mean level of HNF-4A variants in nuclear fractions from three-four independent nuclear fractionation experiments (n = 3–4). ^*^indicates *P* < 0.05. EV = empty vector. WT = wild type. Representative western blots are presented in Supplementary Fig. S2.

Subsequently, we investigated whether the five HNF4A-MODY-associated *HNF4A* variants could disturb normal nuclear localization of HNF-4A (Fig. 3, Table 1). For this, we used transiently transfected HeLa cells. The relative nuclear level was measured after cell fractionation (nuclear/cytosol), SDS-PAGE and immunoblotting using HNF-4A- and nuclear/cytosol marker specific antibodies (Supplementary Fig. S2). Quantification of HNF-4A in nuclear fractions demonstrated strongly reduced levels of R89W (~20%), compared to WT (set to 100%), and increased levels of R85W and R85Q (~240%- and ~160%, respectively). All variants demonstrated exclusively nuclear localization (absent in cytosol) apart from the R89W variant, which was also partially detected in the cytosolic fraction by multiple lower molecular weight bands (Supplementary Fig. S2).

### *HNF4A* variants severely affects HNF-4A DNA binding

**Figure 4:**
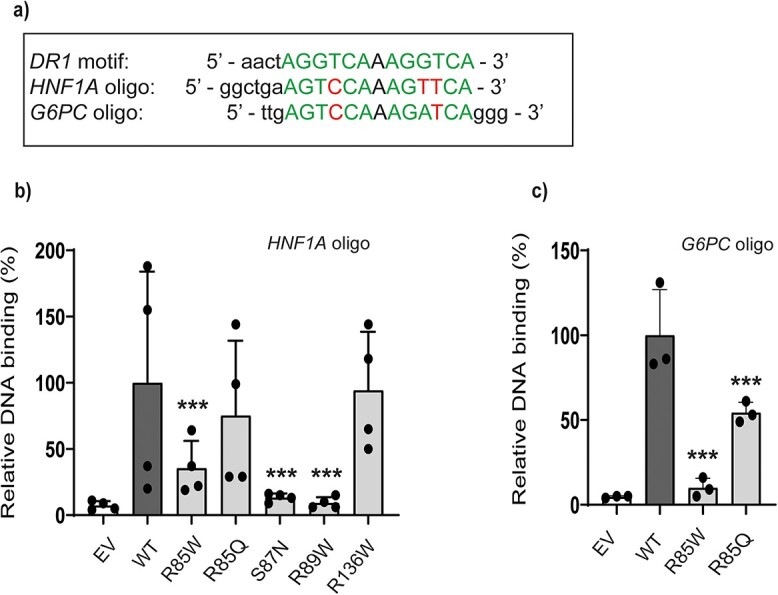
DNA binding of selected *HNF4A* variants by electrophoretic mobility shift analysis (EMSA). (a) HNF-4A recognition site within the *HNF1*A and *G6PC* promoter-based oligo sequences are shown, and relative to the DR1 element consensus recognition site. HNF-4A site base-specific nucleotide contacts made by R85 in *HNF1A* according to [6] and relative to the *G6PC* oligo, are also outlined. HNF-4A variants binding to the (b) *HNF1A* and (c) *G6PC* oligos. Following EMSA, bound complexes were quantified by densitometric analysis. Each bar represents the mean of three independent EMSA experiments (n = 3) ± SD. ^*^^*^indicates *P* < 0.01. EV = empty vector. WT = wild type. Representative gel images are presented in Supplementary Fig. S3.

As the five HNF4A-MODY-associated *HNF4A* variants are located in the HNF-4A DBD, we investigated their effect on HNF-4A binding to an HNF-4A recognition site present in the *HNF1A-* and *G6PC* short oligonucleotide sequences (Fig. 4a, Table 1). All variants except R85Q and R136W demonstrated reduced DNA binding to the recognition site present in the *HNF1A* promoter; DNA binding was severely reduced for variants S87N, R89W and R85W (~13%-, 9% and 35%, respectively) (Fig. 4b, Supplementary Fig. S3a). A supershift assay further verified the presence of HNF-4A variant proteins in the *HNF1A-*oligo bound complexes (Supplementary Fig. S3b). However, none of the variants tested appeared to exert any dominant negative effect on DNA binding by WT HNF-4A (Supplementary Fig. S3c). For a follow-up investigation of the differential effect of the two variants (R85W and R85Q) affecting the same residue (R85), we assessed their binding to the *G6PC* oligonucleotide sequence, for interrogating the consequence of R85 making fewer base pair contacts within the HNF-4A recognition sequence of *G6PC* [6] compared to the *HNF1A* oligo (Fig. 4b). Variant R85W showed significantly reduced binding to the *G6PC* oligo (~10%), similar to the effect on binding to the *HNF1A* oligo, while R85Q, which showed near normal binding to the *HNF1A* oligo, demonstrated only ~50% binding to the *G6PC* oligo.

### R85W and R85Q differentially affect promoter transactivation in different cell lines

**Figure 5:**
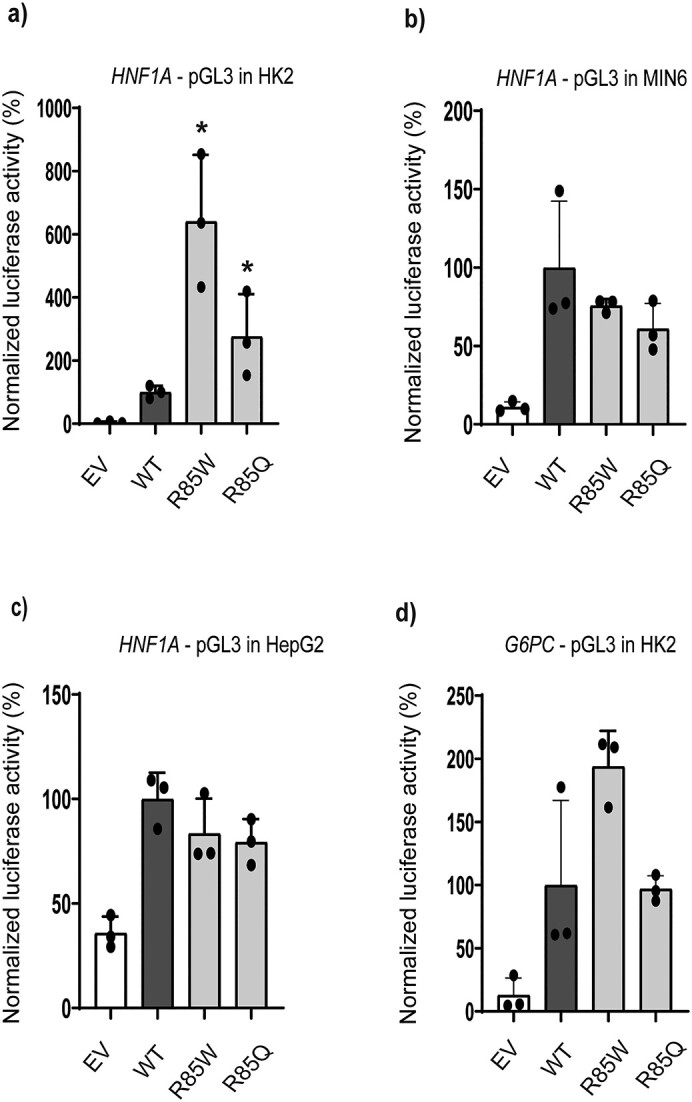
Transactivation by HNF-4A variant on *HNF1A-* and *G6PC*-linked reporter systems in transiently transfected (a and d) HK2, (b) MIN6, and (c) HepG2 cells transfection by WT or *HNF4A* variant plasmids together with (a–c) pGL3-*HNF1A* and (d) pGL3-*G6PC* reporter plasmids. Empty vector (EV) was used as a negative control. Each bar represents the mean of nine readings ± SD, taken from three parallel readings from each of three independent experimental days. ^*^indicates *P* < 0.05.

Intrigued by our findings concerning R85W in particular, which poorly binds the HNF-4A recognition site in the *HNF1A-* and *G6PC* short oligonucleotide sequences (Fig. 4b and c), but demonstrated increased transactivation of the reporters driven by the larger *HNF1A*- and *G6PC* promoter sequences in HeLa and HepG2 cells, respectively (Fig. 2a–c), we aimed to investigate whether R85W activity on these subsequent reporters was also increased in additional relevant cell lines. For this, transactivation was also assessed in HK2, HepG2 and MIN6 cells (Fig. 5 and Table 1). For comparison we included R85Q, which affect the same residue as R85W but had a less deleterious impact in DNA binding and HeLa/HepG2 transactivation assay (Fig. 4b and c, Fig. 2a–c). In transiently transfected HK2 cells (Fig. 5a), R85W significantly increased activity on the *HNF1A* promoter compared to WT (~650% activity) and this increase was greater than that observed in HeLa cells. R85Q also showed increased activity by ~260% compared to WT in HK2 cells, whereas there was no significant difference in HeLa cells. Surprisingly, in HepG2 and MIN6 cells, both R85W and R85Q presented with slightly reduced/near normal activity (~75%–80%) on the *HNF1A* promoter (Fig. 5b and c). The combination of *HNF1A*/HepG2 cells, however, indicates challenges concerning interference of presumably endogenous HNF-4A on the *HNF1A* promoter, shown by higher activity in the EV sample (> 35%) (Fig. 5c), compared to that detected for the HNF1A/HK2 and HNF1A/MIN6 combinations (Fig. 5a and b).

We also investigated variant activity on the *G6PC* promoter-linked reporter in HK2 cells (Fig. 5d). Results indicated similar activity as in HepG2 cells, by increased activity of R85W (~180%) and near normal activity of R85Q. Overall, our transactivation assays indicate a cell line specific effect of variant activity on particularly the *HNF1A* promoter (Fig. 2, Fig. 5). As *G6PC* is not expressed in pancreatic β-cells, transactivation of HNF-4A variants was not assessed in MIN6 cells.

We further wanted to see whether we could confirm the variant specific effect on endogenous *G6PC* and *HNF1A* regulation and expression. For this, RNA was extracted from HK2 cells transfected with expression constructs for HNF-4A variants, and *G6PC* and *HNF1A* expression was investigated by real-time qPCR (Supplementary Fig. S4). A modest increase in *G6PC* expression was detected for R85W (1.7-fold versus WT of 1.6-fold), while a significantly reduced expression of *G6PC* was seen by R85Q (1.4-fold). In contrast, the variants had no significant effect on the expression of endogenous *HNF1A*.

### *In silico* analyses of HNF-4A variants indicate variant induced structural effects

**Figure 6:**
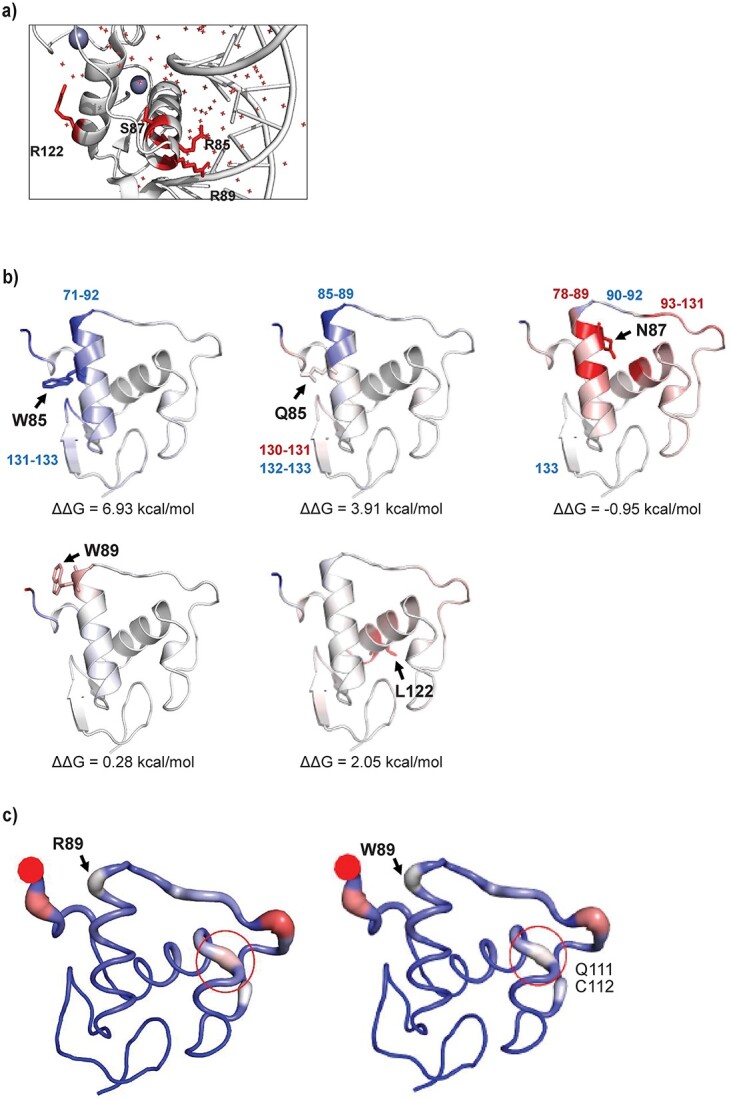
Protein structural changes induced by HNF-4A variant residues. (a) Position of HNF-4A residues subject to change. (b) Protein flexibility change induced by variants according to Eris. Changes in vibrational free energy are indicated by color: Blue indicates increased molecular rigidity, while red indicates increased flexibility compared to WT. R85W: Reduced flexibility of helix residues 71–92 and for Q131-E133. R85Q: Reduced flexibility for helix residues 85–89. S87N: Increased flexibility of helix residues 78–89 and residues M93-Q131, reduced flexibility of L90-H92 and E133. Position of variant residues are shown by arrows. (c) Deformation of protein structure by R89W as predicted by DynaMut. Protein deformation or strain indicated by widening of the molecular backbone and (highlighted circle), reduced strain/deformation predicted at residues Q111 and C112.

*In silico* structural analyses were performed on HNF-4A variants aiming for a structural explanation for variant induced loss-of-function or gain-of-function detected by our *in vitro* functional assays. Various software tools were applied for predicting the residue change effect on the protein flexibility, DNA interaction and binding through available hydrogen bonds, the entropy and energy change, and the stability of the HNF-4A variant protein (Supplementary Tables S2–S5). Only variant residues located within the crystallized protein structure (PDB i.d. 3CBB) were analyzed (Fig. 6a; five of seven variants). Changes in molecular flexibility by residue induced protein structure deformations, as analyzed by Eris, are shown in Fig. 6b. Illustration of residue induced protein structure deformations, as predicted by DynaMut, is shown in Fig. 6c.

Compared to R85, there was strong consensus among the software tools used that W85 was predicted to be stabilizing for the HNF-4A protein structure (Supplementary Table S2). Of all amino acid residues studied, W85 was also predicted to most impact the flexibility (reduced) of the molecular structure (helix residues 71–92 and residues Q131-E133), and the change in free vibrational energy (ΔΔ*S* Vib). A reduction in available hydrogen bonds for DNA-binding by W85 was also predicted, as well as a reduction in free binding energy, compared to R85. Variant residue Q85 (compared to R85) was also predicted to reduce the flexibility of the HNF-4A helix structure from residues 85–89 and residues N132 and E133 (increased for V130 and Q131), as well as reducing the number of hydrogen bonds available for DNA-binding, however not to the extent as W85. Although variant residue W89 most strongly impacted the free binding energy of residue 89 (compared to wild type residue R89), W89 was also predicted to cause a deformation of the molecular structure including residues Q111 and C112 (Fig. 6c). Further, a complete loss of hydrogen bonds was also predicted for W89. The S87 to N87 residue change was not predicted to affect the number of direct hydrogen bonds, as none are in direct contact with DNA, and was rather predicted to result in an additional indirect hydrogen bond. Further, increased molecular flexibility of the protein was predicted for the S87N variant, particularly for residues 78–89 within the same helix as the substitution, and for residues M93 to Q131. Reduced flexibility was predicted for residues L90-H92 and E133. The predicted effects of R85W, R85Q, R89W and S87N on protein structure/stability were also supported by their high REVEL scores of 0.949, 0.921, 0.913 and 0.925, respectively (Supplementary Table S1), and further supporting a likely deleterious impact of the HNF-4A variant.

Although the molecular flexibility was increased for R122, no effect on the free energy of binding was predicted for the L122 to R122 change, supported also by the lower REVEL score of R122L (0.776) compared to the other HNF-4A protein variants investigated by structural *in silico* analyses (Supplementary Table S1). Further, although the L122R substitution was predicted to result in loss of two hydrogen bonds, this amino acid sidechain faces away from DNA in the recognition helix and so the variant has no direct impact on DNA-binding.

## Discussion

Investigation of functional impact of MODY-associated gene variants by *in vitro* functional studies is an important supplement in evaluating the degree of variant-induced protein dysfunction and disease causality, and to disclose clinical actionable variants [19, 20]. The well characterized *HNF1A* gene and HNF-1A protein has largely contributed to this, where variant molecular pathogenicity is defined by sets of threshold values in functional assays and specified by the gene specific ACMG/AMP variant interpretation guidelines by the ClinGen Monogenic Diabetes Expert Panel [21]. According to the Human Gene Mutation Database, 202 disease causing variants (> 115 missense) have so far been reported in the *HNF4A* gene. As only a few variants located in the protein coding region have been functionally studied to date [22], the basis for guiding threshold values for functional evaluation of variant induced HNF-4A dysfunction, for diagnostic purposes, is currently lacking. To tackle this, increased knowledge of the gradient of *HNF4A* variant functional effect is needed. Such studies should be based on robust and sensitive *in vitro* functional assays, in relevant cell line systems, that can distinguish between variants associated with different clinical phenotypes.

The maintenance of glucose homeostasis involves several complementary physiologic processes including glucose resorption (GI tract) and reabsorption (kidneys), glycogenolysis (liver), gluconeogenesis (liver/kidney) and glucose excretion (kidney). In an attempt to capture an *HNF4A* variant effect in multiple systems, our functional HNF-4A investigations were performed in several cell lines, three of which represent cell models from tissues contributing to glucose homeostasis. Further, the *HNF1A* and *G6PC* genes are known HNF-4A targets for DNA binding and transactivation in tissues where HNF-4A plays a central gene regulatory role, like for instance the kidney, liver and pancreas [23].

Our study demonstrates the relevance of the *in vitro* DNA binding assay for determining effects of variants located in the HNF-4A DBD on binding to recognition sites in *HNF1A* and *G6PC* promoters (Fig. 4). In binding assays using the *HNF1A* short oligonucleotide, we observed clear differences between the impact of variants S87N and R89W compared to that of R136W. A severe impact of R85W and R89W variants on binding to the ApoA1 site A probe has previously been reported [24], while we observed varying degrees of reduced binding by variants at R85 to the *G6PC* oligonucleotide. The low DNA binding exhibited by variant S87N is in contrast to a report by Chandra et al. [25], which in a crystal structure of HNF-4A DBD-hinge-LBD multi-domain bound to DNA, identified S87 as facing away from DNA within the recognition helix and thus unable to participate directly in DNA binding, as confirmed by our *in silico* structural analyses. However, S87 has been shown to undergo targeted phosphorylation by protein kinase C, and this modification disrupted the ability of HNF-4A to bind DNA [26]. Residues R85 and R89, on the other hand, have been reported to be in direct contact with the DNA response element AGGTCA half-sites [6, 25]. This is in line with our findings of severely reduced DNA binding by variants R85W and R89W. Our *in silico* analyses also predicted W85 and W89 to dramatically reduce the number of hydrogen bonds (also reduced for Q85, Supplementary Table S4). The reduced binding of variants R85W and R85Q to the *G6PC* oligo compared to the *HNF1A* oligonucleotide could be a consequence of R85 making three base pair contacts within the HNF-4A recognition site of the *HNF1A* oligo, versus two in the *G6PC* oligo [6]. The near-normal DNA binding of R136W to *HNF1A* is, however, somewhat in contrast to previous reports investigating binding to *DR1/HNF1A* sequences, using purified HNF-4A protein or from nuclear extracts [25, 27]. Although R136W was not assessed for *G6PC* binding in the DNA binding assay, a loss-of-transactivation function (< 50%) was, however, found on the *G6PC*-reporter in HepG2 cells (Fig. 2). This could suggest a potential stronger variant effect on *G6PC-* compared to *HNF1A* transactivation. R136 has further been reported to lie in a sensitive domain-domain junction, forming domain-domain arrangements with the upstream DBD in the HNF-4A crystal structure [25].

HeLa cells have been shown to be a suitable cell line for studying the functional consequence of *HNF1A* variants on HNF-1A transactivation and for successfully distinguishing pathogenic variants from benign and T2D associated variants [28]. In our study, a functional discrimination between severe (HNF4A-MODY linked) and milder (T2D associated) *HNF4A* variants on HNF-4A transactivation (*HNF1A* reporter) in HeLa cells was less evident and clouded by substantial intra-variant variations. Use of the *G6PC*- linked reporter system in HepG2 cells, however, was more successful in distinguishing between near-normal, loss- and gain-of-transactivation function variants (Fig. 2). The near-normal activity of the T2D associated variant T139I is in line with that reported elsewhere on *HNF1A*- or *TK*-linked reporter constructs [29, 30]. The high transactivation activity of R85W on both *G6PC*- and *HNF1A* promoter-linked reporters in HepG2 and HK2 cells (partly also for R85Q variant), is intriguing (Figs 2 and 5), particularly considering the low DNA binding ability (Fig. 4). It is important thus to bear in mind the differences in the size of the promoter-specific DNA sequences used in the DNA binding versus transactivation assays. The interaction of HNF-4A with other promoter-bound factors could potentially allow recruitment of HNF-4A to the promoter-linked transactivation reporters even in the presence of reduced direct binding to DNA. A contribution of potential HepG2 and HK2 cell-specific coactivators to the increased R85W promoter activity would, however, require more detailed investigations in a future study. Worth noting, however, is that in HepG2 cells, the peroxisome proliferator-activated receptor (PPAR)γ coactivator 1-alpha (PGC-1α) has been shown to coactivate HNF-4A, and that the presence of adjacent RORα/SRC-2 is necessary for the full transcriptional effect of PGC-1α [31]. Whether R85W, predicted to stabilize HNF-4A and reduce the flexibility of the molecular structure, forms a more stable interaction with PGC-1α in the HNF-4A C-terminal AF-II domain, is unknown. In addition to HNF-4A, these cofactors are also reported to be expressed at high levels in kidney tissue (the Human Protein Atlas).

Further, W85 was predicted to increase the rigidity and decrease the flexibility of helix 1 by our *in silico* analyses. In the absence of crystal structures of the HNF-4A AF-I and repressor domains, it is however impossible to predict how a putative increase in rigidity due to the R85W substitution might alter the potency of the AF-I domain activity function, or alternatively whether the increased activity of the variant results from an effect on the inhibitory function of the HNF-4A repressor domain [4, 32].

HNF-4A has been shown to play an important role in the upregulation of membrane transporters and receptor proteins for the reabsorption of various molecules in kidney proximal tubular epithelial cells [33], whereby the transport of glucose in and out of cells is mediated by specialized transport proteins [34]. Glucosuria, which is more frequent in HNF1A-MODY patients, is a biochemical feature also reported in R85W carriers [10]. Glucosuria may be due to a disturbance in glucose resorption or release by proximal tubular cells. The proximal tubule generates glucose-6-phosphate from various precursors, and through glucose-6-phosphatase subsequently generates free glucose that can exit the cell. The increased activity of R85W on the *G6PC*-linked reporter in HK2 cells (Fig. 5) may suggest increased regulation of glucose-6-phosphatase and increased glucose release by kidney tubules (renal gluconeogenesis is projected to potentially be responsible for approximately 40% of all gluconeogenesis [35]). Further, increased activity of R85W on the same promoter in HepG2 cells (Fig. 2) may also suggest a mechanism of increased output of glucose from the liver.

There is much evidence for a cross-regulatory loop taking place between HNF-1A and HNF-4A in pancreatic cells [35, 36]. Stimulated circuits, very much like the HNF-1A/HNF-4A loop, have been previously shown to exhibit bistability [37, 38], where loss of one functional allele can increase the probability that the opposite gene is inhibited sufficiently to trigger the transition to an “OFF” state. Whether the mildly reduced R85W and R85Q activity on the *HNF1A*-linked reporter in MIN6 cells (Fig. 5) is sufficient to affect the bistability of such an HNF-1A/HNF-4A loop circuit in β-cells, causing reduced expression of HNF-1A and HNF-4A regulated downstream genes, requires more detailed investigations. Further, whether the biphasic phenotype of HH followed by HNF4A-MODY in association with the R85W variant could also be due to a contribution of increased gluconeogenesis in the kidneys and glycogenolysis from the liver in the post-absorptive state, is an interesting hypothesis that would also require further detailed investigations.

Our nuclear localization assay successfully separated variants with severely reduced nuclear levels (R89W, present at 20% of the normal level) from those with near-normal or increased levels (R85W and R85Q) (Fig. 3). The affected residue R89 has been reported to lie within the nuclear localization signal (NLS) of HNF-4A (residues 80–121), which also contains a site for acetylation and is important for proper nuclear retention [39]. Although our *in silico* analyses were inconclusive regarding a destabilizing effect of the R89W substitution (Supplementary Table S2), W89 was predicted to cause a deformation of the molecular structure of nearby residues, which might contribute to reduced levels of protein (Fig. 6). However, while NLSs are typically rich in lysine and arginine residues, the efficient nuclear localization observed for the R85W and R89W variants suggest that the effect of R89W is not simply due to a reduction in positive charge. Finally, the normal nuclear localization of R136W correlates with previous reports [27].

Since the criteria for evaluating variant molecular pathogenicity by functional assays, set by the ClinGen Monogenic Diabetes Expert panel and *HNF4A* specific AMCG/AMP guidelines, are directed towards loss-of-function variants, these guidelines are not applicable for interpretation of variants displaying assay specific gain-of-function (e.g. R85W and partly R85Q). Our functional data however supports T139I representing a benign variant since it qualifies for the use of BS3_supporting criteria according to the thresholds set by the *HNF4A* specific ACMG guidelines and R89W representing a pathogenic variant qualifying for the use of PS3_supporting criteria. Moreover, our functional data also supports pathogenicity for the S87N variant (PS3_supporting), however this variant still remains a VUS according to the ACMG guidelines (Table 1, Supplementary Table S1).

In conclusion, we recommend the use of the *G6PC*-linked reporter system in HepG2 cells to best capture the gradient of functional effect of *HNF4A* diabetes-associated variants. Moreover, as MODY gene variant investigations require appropriate internal controls for evaluating variant pathogenic effects by functional assays, our study identified the following control variants for studies in HepG2 cell transactivation assay; S87N for severely reduced activity (< 50%) and T139I for near-normal activity. Further, variant R89W is proposed as control variant for severely reduced nuclear localization in HeLa cells (< 25%), and S87N/R89W as control variants for DNA binding using the *HNF1A* oligo (< 20%). The systematic use of the same control variants across laboratories in functional investigations of *HNF1A* variants has proven extremely important for capturing the range of variant effects across the *HNF1A* allelic spectrum. This has allowed recommendations of assay-specific threshold values for diagnostic purposes, defining what is “decreased” and “normal” function, and should also be implemented for future *HNF4A* variant investigations.

## Materials and methods

### *HNF4A* gene variants

Seven previously reported *HNF4A* missense variants (R85W, R85Q, S87N, R89W, R122L, R136W and T139I) associated with different glycemic phenotypes in variant carriers were selected for functional evaluation [15–18]. Their domain location, ClinVar and REVEL scores, and associated clinical phenotype, are shown in Supplementary Table S1. Further, *HNF4A* variant interpretation was performed according to the ClinGen Monogenic Diabetes Expert Panel specifications to the American College of Medical Genetics (ACMG) and Genomics/the Association for Molecular Pathology (AMP) guidelines for *HNF4A* variants, except for variant R136W which due to its reduced penetrance could not be classified by the standard ACMG guidelines. The PS3_supporting or BS3_supporting ACMG scores were only applied when a variant was shown to be functionally impaired (transactivation activity < 60% and DNA binding < 60%, compared to wild type) or showed no damaging effect (transactivation activity > 75% compared to wild type), respectively, in at least two functional assays by transactivation and/or DNA binding assay. The nomenclature used for the variants is based on the *HNF4A* transcript NM_000457.4 (HNF4a isoform2).

### Constructs

All *HNF4A* variants were constructed using the QuikChange II XL Site-Directed Mutagenesis Kit (Agilent Technologies) and variant-specific primers (sequences available upon request). Individual *HNF4A* variants were introduced into the WT *HNF4A* cDNA isoform 2 (NCBI NM_000457.4), in FLAG-tagged pcDNA3.1+ vector. In the transactivation assays, the following reporter plasmids coupled to *Firefly luciferase* by *luc +* in pGL3 (Promega) were used: the human *HNF1A* gene promoter sequence (nucleotides −129 to +196) kindly provided by K. Yamagata (Osaka University, Japan) [27] or the mouse *G6PC* gene promoter sequence (nucleotides −231 to +66) kindly provided by B.W. O’Malley (Baylor College of Medicine, USA), and the *Renilla luciferase* control vector pRL-SV40 (Promega).

### Cell lines and transfection

HeLa cells (human cervical carcinoma cells) were cultured in DMEM medium (without pyruvate) with 10% FBS. HepG2 cells (human hepatoma cells) were cultured in EMEM medium (with pyruvate) with 10% FBS and L-glutamine (290 mM). HK2-cells (human proximal tubule cells) were cultured in Keratinocyte Serum Free Medium (K-SFM) with 0.05 mg/ml Bovine pituitary extract and 5 ng/ml human recombinant Epidermal growth factor. MIN6 cells (mouse insulinoma cells) were cultured in DMEM medium with 15% FBS. All media were supplemented with penicillin/streptomycin (100 U/ml) and cell lines cultured at 37°C with 5% CO_2_ in a humid atmosphere. For transfection purposes, HeLa, MIN6 and HK2 cells were transfected using Lipofectamine2000 (Invitrogen), while HepG2 cells were transfected using the TransIT-LT1 transfection reagent (Mirus Bio).

### Luciferase assays and protein abundance

Cells were transiently transfected with wild-type (WT) or variant *HNF4A* plasmids, together with a *Firefly* reporter plasmid and the *Renilla* reporter plasmid. Luciferase activities were measured 24 h post transfection using the Dual-Luciferase Assay System (Promega) on a Centro XS3 LB 960 luminometer (Berthold Technologies). HNF-4A activity was normalized to *Firefly* and *Renilla* activity. The level of HNF-4A protein expression (corresponds to the canonical protein sequence P41235-1 in the UniProtKB entry for HNF-4A) of WT and HNF-4A variants was assessed in various cell lysates obtained for the transactivation assays. In short, 20 μl of cell lysates was subjected to SDS-PAGE and immunoblotting using specific antibodies against HNF-4A (Cell Signaling; #C11F12) and β-actin (Santa Cruz; sc-47778).

### Subcellular fractionation

Nuclear and cytosolic fractions were isolated from transiently transfected HeLa cells as previously described [40]. Total protein quantification in each fraction was measured using the Pierce™ BCA Protein Assay Kit (Thermo Fisher) and 8–10 μg total protein from fractions was subjected to SDS-PAGE and immunoblotting using rabbit anti-HNF-4A (Cell Signaling; #C11F12). The purity of the fractions was verified using antibodies against the nuclear marker protein Topoisomerase II-α (Cell signaling; #12286) and the cytosolic marker protein GAPDH (Santa Cruz; sc-47724). The relative subcellular localization based on each fraction was determined by calculating the ratios of HNF-4A with the respective nuclear or cytosolic markers.

### DNA binding assay

The electrophoretic mobility shift assay (EMSA) was carried out as previously described [36]. Briefly, equal amounts of proteins from nuclear fractions of transiently transfected HeLa cells were incubated together with cyanine 5-labelled oligonucleotides (Sigma Aldrich) and the binding reaction was performed using the Odyssey EMSA buffer kit (LI-COR Biosciences). The double stranded DNA fragments containing the HNF-4A binding site in the promoter of the *G6PC* gene (5′-TTGAGTCCAAAGATCAGGG-3′) or the HNF-4A binding site in the promoter of the *HNF1A* gene (5′-GGCTGAAGTCCAAAGTTCA-3′) were used in the binding reactions. Bound complexes were analyzed by electrophoresis on 6% (w/v) polyacrylamide gels for EMSA (Thermo Fisher).

### RNA isolation, reverse transcription and real-time quantitative PCR (RT-qPCR)

Total RNA from HK2 cells, transiently transfected with WT or *HNF4A* variant plasmids, was extracted using the PureLink RNA Mini Kit (Thermo Fisher). RNA (300 ng) was reverse transcribed using the qScript™ cDNA Synthesis Kit (Quantabio, Beverly, MA). The effect of transfected *HNF4A* variants on target gene expression was analyzed by RT-qPCR using gene specific primers (sequences available upon request) and SYBRGreen Fast Mix kit (Thermo Fisher). Analysis was performed using theStepOnePlus™ Real-Time PCR system instrument (Thermo Fisher). The reference gene used was *RPL13*.

### Structural analyses

Structural protein modelling of HNF-4A variants was carried out using the crystal structure of the DNA binding domain (residues 33–113; PDBC: 3CBB) and the side-chain rotamer library incorporated within the mutagenesis wizard of the PyMOL version 2.2.2 software (Schrödinger, Inc.). PyMOL, thus, also predicts hydrogen bond status of residues. DynaMut was used to predict the effect of variants on protein flexibility, interaction and stability, by the change in entropy (ΔΔ*G*) and vibrational energy (ΔΔ*S*) of the molecule (Supplementary Table S3) [41]. Eris, which is a protein stability prediction tool, calculates the change ΔΔ*G* using the Medusa Modelling suite, and also models backbone flexibility [42]. Dezyme evaluates changes in the free energy of folding of the variant protein, as well as solvent accessibility [43]. MUpro [44] and CUPSAT [45] are predictors of changes in protein stability caused by missense variants. PreHot uses binding energy calculations to predict hotspots of regions crucial for the interaction between protein and DNA (Supplementary Table S5) [46]. SAMPDI predicts protein variant effect on DNA binding properties, also based on free energy of binding [47].

### REVEL analysis

The Alamut Software (version 2.15, SOPHiA GENETICS) was used to extract REVEL scores for the HNF-4A variants. REVEL [48] is an ensemble method for predicting the damaging effect of missense variants to protein structure or function based on combining scores from 13 individual *in silico* tools: MutPred, FATHMM v2.3, VEST 3.0, SIFT, PolyPhen-s, PROVEAN, MutationTaster, MutationAssessor, LRT, GERP++, phyloP, SiPhy, and phastCons. REVEL scores range from 0 to 1, with higher scores reflecting greater likelihood that a variant is damaging/pathogenic (> 0.7).

### Statistical analysis

All data are presented as mean ± standard deviation (STD) relative to WT (set as 100% or 1-fold), unless otherwise specified. Each experiment was carried out on three independent experimental days unless otherwise specified. Differences between the studied variants were analyzed using GraphPad Prism Software (version 8.1.1) and raw data (i.e. *Firefly*/*Renilla* ratios). Statistical analyses were performed using a 1-way ANOVA and Dunnett’s posthoc test (alpha level of 0.05).

##  

*Conflict of interest statement:* The authors have no conflicts of interest to declare.

## Funding

This study (L.B.) was supported by grants from the Norwegian Diabetes Association (grant #6000026) and from the Western Norway University of Applied Sciences. P.R.N. was ssupported by grants from the European Research Council (AdG #293574), Stiftelsen Trond Mohn Foundation (Mohn Center of Diabetes Precision Medicine), the University of Bergen, Haukeland University Hospital, the Research Council of Norway (FRIPRO grant #240413), the Western Norway Regional Health Authority (Strategic Fund “Personalised Medicine for Children and Adults”), and the Novo Nordisk Foundation (grant #54741). Special acknowledgement and thanks to Sandra Mohr for assisting in structural analyses.

## Supplementary Material

Supplementary_Data_Kaci_ddae027
