# Supplementary material for: Functional characterization of HNF4A gene variants identify promoter and cell line specific transactivation effects
Source: Hum Mol Genet. 2024 Mar 3;33(10):894–904. doi: 10.1093/hmg/ddae027 (PMC11070132; doi:10.1093/hmg/ddae027)
Supplement: Supplementary_Data_Kaci_ddae027 [file supplementary_data_kaci_ddae027.pdf]

Supplemental Data, Kaci et al.

Supplementary Figures

Figure S1: Relative protein expression level of HNF-4A variants in transiently transfected HeLa and HepG2 cells

Figure S2: Assessment of nuclear localization of HNF-4A protein variants.

Figure S3. Binding of wild-type (WT) and variant HNF-4A to the *HNF1A* promoter oligos

Figure S4. Effect of HNF-4A variants on endogenous *G6PC* and *HNF1A* mRNA expression in HK2 cells

Supplementary Tables

Table S1. Classification of *HNF4A* variants

Table S2. Prediction of changes in entropy (ΔΔ*S*) and protein stability (ΔΔ*G*) in missense variants compared to wild-type (WT) by DynaMut, Dezyme, Eris and CUPSAT prediction tools

Table S3. DynaMut predictions for changes in free energy of variants compared to wild-type (WT).

Table S4. The number of hydrogen bonds of the predicted rotamers of WT versus variant residues as predicted by PyMOL.

Table S5. Changes in free energy of binding of residues (to DNA) induced by amino acid changes as predicted by PreHot.

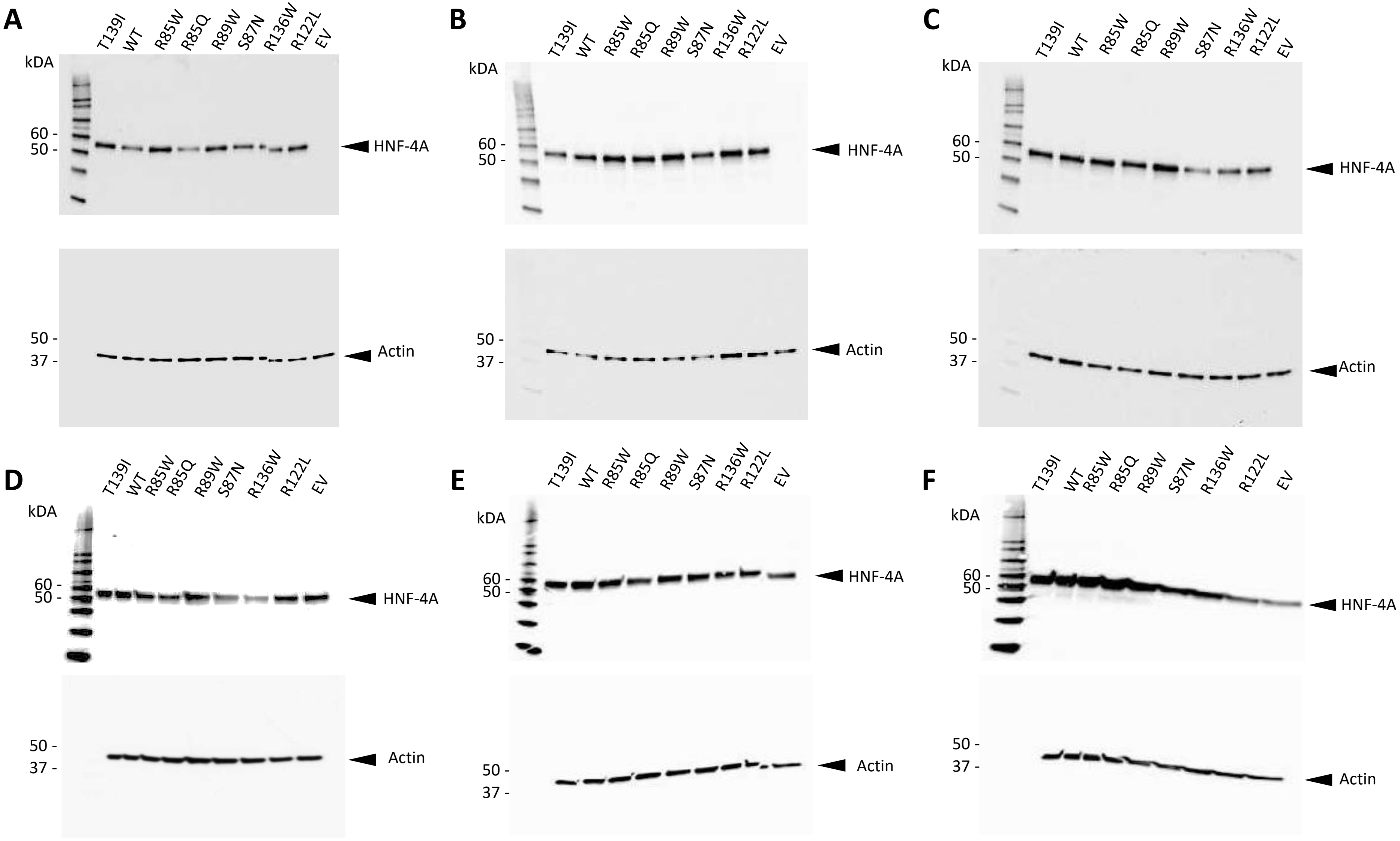

Figure S1. Relative protein expression level of HNF-4A protein variants in transiently transfected HeLa **(a-c)** and HepG2 **(d-f)** cells, analyzed by SDS-PAGE and immunoblotting using HNF-4A and actin specific antibodies. Representative immunoblots from three independent experimental days (n=3).

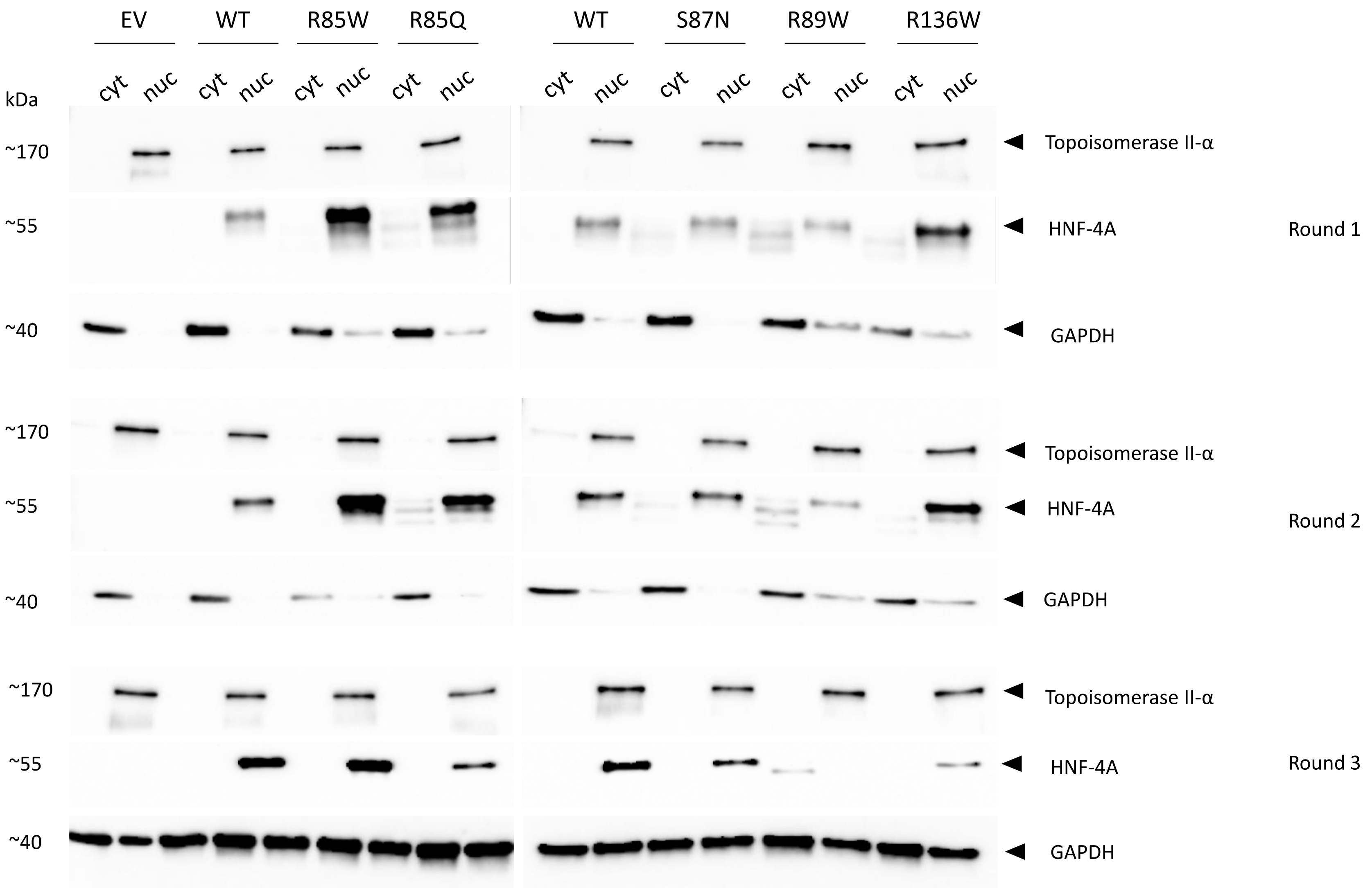

Figure S2. Assessment of nuclear localization of HNF-4A protein variants. HeLa cells were transiently transfected with wild-type (WT) or variant *HNF4A* plasmids, fractionated, and analyzed for HNF-4A and nuclear and cytosolic markers (topoisomerase and GAPDH, respectively) by SDS-PAGE and immunoblotting using appropriate antibodies. Round 1-3 refers to three independent experimental days (n=3).

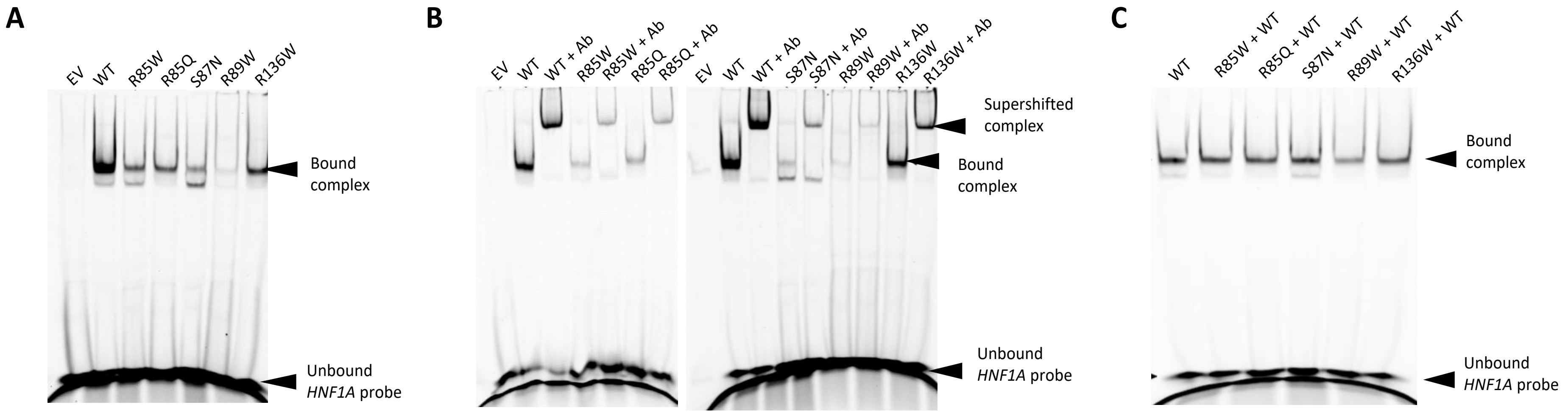

Figure S3. Binding of wild-type (WT) and variant HNF-4A to the *HNF1A* promoter oligos. **a)** Representative image from one of three individual experiments is shown. **b)** Supershift assay showing binding of HNF-4A antibody to the DNA-HNF-4A complex. **c)** Effect of variant on WT HNF-4A DNA binding showing no indication of dominant negative effect of variants.

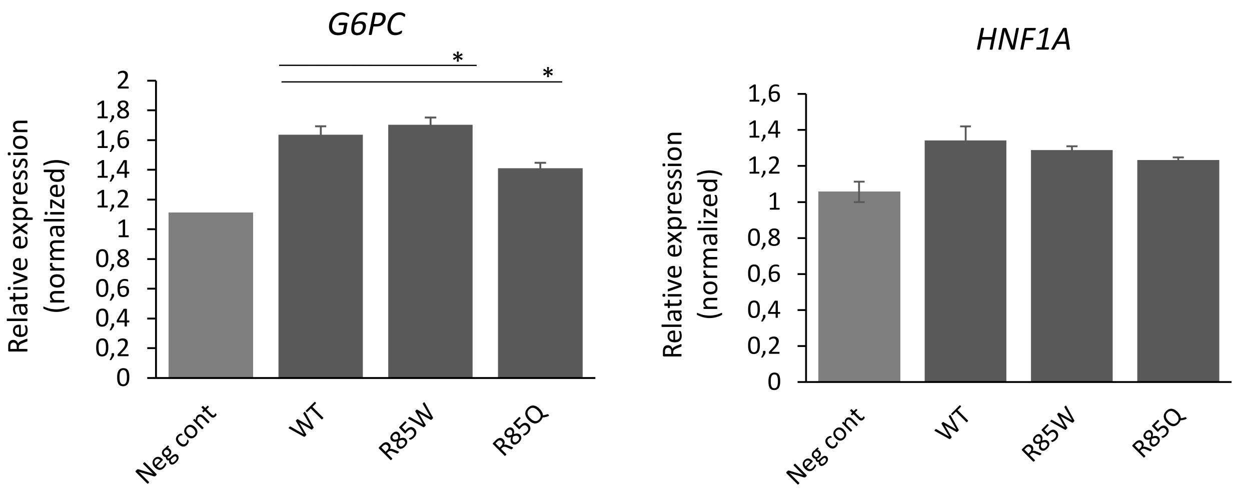

Figure S4. Effect of HNF-4A variants on endogenous *G6PC* and *HNF1A* mRNA expression in HK2 cells. RNA was isolated from HK2 cells transiently transfected with plasmids for expression of wild-type (WT) or variant *HNF4A* and analyzed by qPCR using target gene specific primers. *RPL13* was used as reference gene and for normalization of Ct values of target genes. Each bar represents the mean fold change ±SD of three biological replicates (n=3). *indicates $p<0.05$.

Table S1. Classification of *HNF4A* variants.Rare Exome Variant Ensemble Learner (REVEL) score for an individual missense variant can range from 0 to 1 (1 is the highest score correlating with a greater likelihood of that variant being disease-causing)(1). *HNF4A* variant interpretation was performed according to the ClinGen Monogenic Diabetes Expert Panel specifications to the American College of Medical Genetics (ACMG) and Genomics/the Association for Molecular Pathology guidelines (2) (ClinGen Monogenic Diabetes Expert Panel Specifications for *HNF4A*, v 1.1.0, 2023 (3), except for variant p.Arg136Trp which due to its reduced penetrance could not be classified by the standard ACMG guidelines.

**Nucleotide/ protein variant (HNF4alpha2 isoform) (NM_000457.4)**	**Nucleotide/ protein variant (HNF4alpha8 isoform) (NM_175914.5)**	**Other nomenclature** (Chartier, Bossu et al. 1994)	**Functional domain**	**ClinVar**	**ACMG classification[2,3]**	**REVEL score[1]**	**Associated with phenotypes**
c.253C>T p.(Arg85Trp)	c.187C>T p.(Arg63Trp)	p.(Arg76Trp)	DBD	Px11	*Pathogenic* (PS4, PP1 strong, PS2_mod, PM1, PM2_sup, PM5_sup, PP3, PP4)	0.949	Fanconi renotubular syndrome 4 with MODY
c.254G>A p.(Arg85Gln)	c.188G>A p.(Arg63Gln)	p.(Arg76Gln)	DBD	LP	*Likely pathogenic* (PM1, PS4_mod, PM2_sup, PM5_sup, PP3)	0.921	Fanconi syndrome and MODY
c.260G>A p.(Ser87Asn)	c.194G>A p.(Ser65Asn)	p.(Ser78Asn)	DBD	No	*VUS* (PM1_sup, PM2_sup PP3)	0.913	MODY
c.265C>T p.(Arg89Trp)	c.199C>T p.(Arg67Trp)	p.(Arg80Trp)	DBD	No	*Pathogenic* (PP1_strong, PS4_mod, PM1, PM2_sup, PM5_sup, PP3)	0.925	Hyperinsulinemic hypoglycemia and MODY
c.365G>T p.(Arg122Leu)	c.299G>T p.(Arg100Leu)	p.(Arg113Leu)	DBD	No	*VUS* (PM1_sup, PM2_sup, PP3)	0.776	T2D
c.406C>T p.(Arg136Trp)	c.340C>T p.(Arg114Trp)	p.(Arg127Trp)	Hinge	VUSx3 LPx1 Px4	*N/A*	0.919	MODY (reduced penetrance)
c.416C>T p.(Thr139Ile)	c.350C>T p.(Thr117Ile)	p.(Thr130Ile)	Hinge	Bx5 LBx3	*Benign* (BA1)	0.426	T2D

Abbreviations: DBD, DNA binding domain; B, Benign; LB, Likely Benign; VUS, Variant of Uncertain Significance; Likely Pathogenic, LP; P, Pathogenic; mod, moderate; sup, supportive; N/A, Not Applicable.

1. Ioannidis, NM., Rothstein, J.H., Pejaver, V., Middha, S., McDonnell, S.K., Baheti, S., Musolf, A., Li, Q., Holziger, E., Karyadi, D., et al. (2016) REVEL: An Ensemble Method for Predicting the Pathogenicity of Rare Missense Variants. *Am J Hum Genet*, **99**, 877-885.
2. Richards, S., Aziz, N., Bale, S., Bick, D., Das, S., Gastier-Foster, J., Grody, W.W., Hegde, M., Lyon, E., Spector, E. et al. (2015) Standards and guidelines for the interpretation of sequence variants: a joint consensus recommendation of the American College of Medical Genetics and Genomics and the Association for Molecular Pathology. *Genet Med*, **17**, 405-424.
3. Monogenic Diabetes VCEP (2023) ClinGen Monogenic Diabetes Expert Panel Specifications to the ACMG/AMP Variant Interpretation Guidelines for HNF4A Version 1.1.0.

Table S2. Prediction of changes in entropy (ΔΔ*S*) and protein stability (ΔΔ*G*) in missense variants compared to wild-type (WT) by DynaMut, Dezyme, Eris and CUPSAT prediction tools.

Method	R85Q ΔΔG [kcal/mol]	Type	R85W ΔΔG [kcal/mol]	Type	S87N ΔΔG [kcal/mol]	Type	R89W ΔΔG [kcal/mol]	Type	R122L ΔΔG [kcal/mol]	Type
**Dynamut**	0,05	stabilizing	0,91	stabilizing	0,12	stabilizing	0,28	stabilizing	0,24	stabilizing
**Dezyme**	0,45	stabilizing	0,75	stabilizing	1,05	stabilizing	0,87	stabilizing	0,13	stabilizing
**Eris**	3,91	stabilizing	6,93	stabilizing	-0,95	destabilizing	9,11	stabilizing	2,05	stabilizing
**CUPSAT**	-0,86	destabilizing	1,31	stabilizing	0,61	stabilizing	-0,2	destabilizing	0,43	stabilizing

Table S3. DynaMut predictions for changes in free energy of variants compared to wild-type (WT).

Variant	ΔΔS Vib	ΔΔG ENCoM	ΔΔG mCSM	ΔΔG SDM	ΔΔD DUET	ΔΔG	Type
**Q85**	-0,09	0,07	-1,07	-0,33	-0,85	0,05	Stabilizing
**W85**	-0,44	0,36	-0,60	0,06	-0,60	0,91	Stabilizing
**N87**	0,19	-0,15	-0,34	0,33	0,10	0,12	Stabilizing
**W89**	0,00	0,00	-0,19	0,59	-0,16	0,28	Stabilizing
**L122**	0,14	-0,11	0,45	-0,08	0,56	0,24	Stabilizing

Table S4. The number of hydrogen bonds of the predicted rotamers of wild-type (WT) versus variant residues as predicted by PyMOL.

Residue	Probability (%)	Direct bonds	Indirect bonds	vNA	Hydrogen bonds
**R85 (WT)**		0	7	0	9
**Q85**-R1	20,9	0	0	0	1
**Q85**-R2	14,4	0	1	0	2
**Q85**-R3	13,6	0	2	1	3
**R85 (WT)**		0	7	0	9
**W85**-R1	37,0	0	0	0	0
**W85**-R2	30,2	0	0	0	0
**W85**-R3	10,6	1	0	0	1
**S87 (WT)**		0	0	0	2
**N87**-R1	51,0	0	1	0	2
**N87**-R2	12,2	0	0	0	2
**R89 (WT)**		2	0	0	6
**W89**-R1	31,9	0	0	0	0
**W89**-R2	21,0	0	0	0	0
**W89**-R3	14,6	0	0	0	1
**W89**-R4	14,4	0	0	0	1
**R122 (WT)**		0	0	0	2
**L122**-R1	57,0	0	0	0	0
**L122**-R2	37,2	0	0	0	0

The bonds are separated into Direct bonds (hydrogen bonds directly from the amino acid to a nucleic acid), Indirect bonds (a water mediated hydrogen bond between an amino acid and a nuclei acid), vNA (bonds from one amino acid to another and to a nucleic acid), and Hydrogen bonds (the total number of hydrogen bonds the amino acid forms). R, rotamers.

Table S5. Changes in free energy of binding of residues (to DNA) induced by amino acid changes as predicted by PreHot.

Variant	R85	R86	S87	R89	N110	R116	R122
**R85**	0,12	0,51	None	0,11	0,81	0,16	None
**Q85**	0,14	0,46	None	0,45	0,16	0,54	None
**W85**	None	0,46	None	0,45	0,16	0,54	None
**N87**	0,12	0,46	None	0,48	0,16	0,54	None
**W89**	0,13	0,47	None	0,94	0,16	0,54	None
**L122**	0,12	0,46	None	0,45	0,16	0,54	None
